# A blood transcriptome-based analysis of disease progression, immune regulation, and symptoms in coronavirus-infected patients

**DOI:** 10.1038/s41420-020-00376-x

**Published:** 2020-12-08

**Authors:** Anguraj Sadanandam, Tobias Bopp, Santosh Dixit, David J. H. F. Knapp, Chitra Priya Emperumal, Paschalis Vergidis, Krishnaraj Rajalingam, Alan Melcher, Nagarajan Kannan

**Affiliations:** 1 Division of Molecular Pathology, The Institute of Cancer Research, London, UK; 2 Division of Experimental Pathology, Department of Laboratory Medicine and Pathology, Mayo Clinic, Rochester, MN 55905 USA; 3 Institute for Immunology, University Medical Center, Johannes Gutenberg University Mainz, Mainz, Germany; 4 Centre for Translational Cancer Research (CTCR; a joint initiative of Indian Institute of Science Education and Research (IISER) Pune and Prashanti Cancer Care Mission), Pune, India; 5 Institut de recherche en immunologie et en cancérologie, Université de Montréal, Montreal, QC Canada; 6 Département de pathologie et biologie cellulaire, Université de Montréal, Montreal, QC Canada; 7 Herman Ostrow School of Dentistry, University of Southern California, Los Angeles, CA USA; 8 Division of Infectious Diseases, Mayo Clinic, Rochester, MN USA; 9 Cell Biology Unit, University Medical Center of the Johannes Gutenberg University Mainz, Mainz, Germany; 10 University Cancer Center Mainz, University Medical Center, Mainz, Germany; 11 Division of Radiotherapy and Imaging, The Institute of Cancer Research, London, UK; 12 Center for Regenerative Medicine, Mayo Clinic, Rochester, MN 55905 USA; 13 Mayo Clinic Cancer Center, Mayo Clinic, Rochester, MN 55905 USA

**Keywords:** Immunology, Molecular biology

## Abstract

COVID-19 patients show heterogeneity in clinical presentation and outcomes that makes pandemic control and strategy difficult; optimizing management requires a systems biology approach of understanding the disease. Here we sought to potentially understand and infer complex disease progression, immune regulation, and symptoms in patients infected with coronaviruses (35 SARS-CoV and 3 SARS-CoV-2 patients and 57 samples) at two different disease progression stages. Further, we compared coronavirus data with healthy individuals (*n* = 16) and patients with other infections (*n* = 144; all publicly available data). We applied inferential statistics (the COVID-engine platform) to RNA profiles (from limited number of samples) derived from peripheral blood mononuclear cells (PBMCs). Compared to healthy individuals, a subset of integrated blood-based gene profiles (signatures) distinguished acute-like (mimicking coronavirus-infected patients with prolonged hospitalization) from recovering-like patients. These signatures also hierarchically represented multiple (at the system level) parameters associated with PBMC including dysregulated cytokines, genes, pathways, networks of pathways/concepts, immune status, and cell types. Proof-of-principle observations included PBMC-based increases in cytokine storm-associated *IL6*, enhanced innate immunity (macrophages and neutrophils), and lower adaptive T and B cell immunity in patients with acute-like disease compared to those with recovery-like disease. Patients in the recovery-like stage showed significantly enhanced *TNF*, *IFN-γ*, anti-viral, *HLA-DQA1*, and *HLA-F* gene expression and cytolytic activity, and reduced pro-viral gene expression compared to those in the acute-like stage in PBMC. Besides, our analysis revealed overlapping genes associated with potential comorbidities (associated diabetes) and disease-like conditions (associated with thromboembolism, pneumonia, lung disease, and septicemia). Overall, our COVID-engine inferential statistics platform and study involving PBMC-based RNA profiling may help understand complex and variable system-wide responses displayed by coronavirus-infected patients with further validation.

## Introduction

The spread of COVID-19, a disease caused by severe acute respiratory syndrome coronavirus 2 (SARS-CoV-2), has led to the current global pandemic with already millions of people with confirmed infection and more than one million deaths^1^. According to the World Health Organization (WHO), the mode of infection for COVID-19 is predominantly through respiratory droplets, aerosol transmission due to pathogen-laden viral particles in the air, or close contact with infected people with increased viral loads, especially in the early stages of disease^2^. The mechanism of human pathogenesis, to a great extent, may simulate that of SARS-CoV (associated with SARS) and Middle East respiratory syndrome coronaVirus (MERS-CoV; associated with MERS) viral infections, including the prolonged persistence of the virus worsening the host immune response^3–5^. Clinical manifestation of COVID-19 ranges from mild respiratory symptoms to severe disease and death^6^. However, there are now reports suggesting heterogeneous manifestation of the disease affecting multiple organs, including kidney, liver, and brain^7^. Although age and compromised health history are considered critical prognostic factors, certain patients of younger age and good health have shown severe progression of the disease^8^.

Patients with COVID-19 may be asymptomatic^9,10^, but can still transmit infection^11^. Viral shedding from an infected person may occur, although resolution of symptoms^12^, and relapse has been reported despite consecutive negative testing^13^. Currently, there are no approved therapies for COVID-19 respiratory symptoms^14,15^ or way to screen for disease progression, and system-wide changes in patients.

**Fig. 1:**
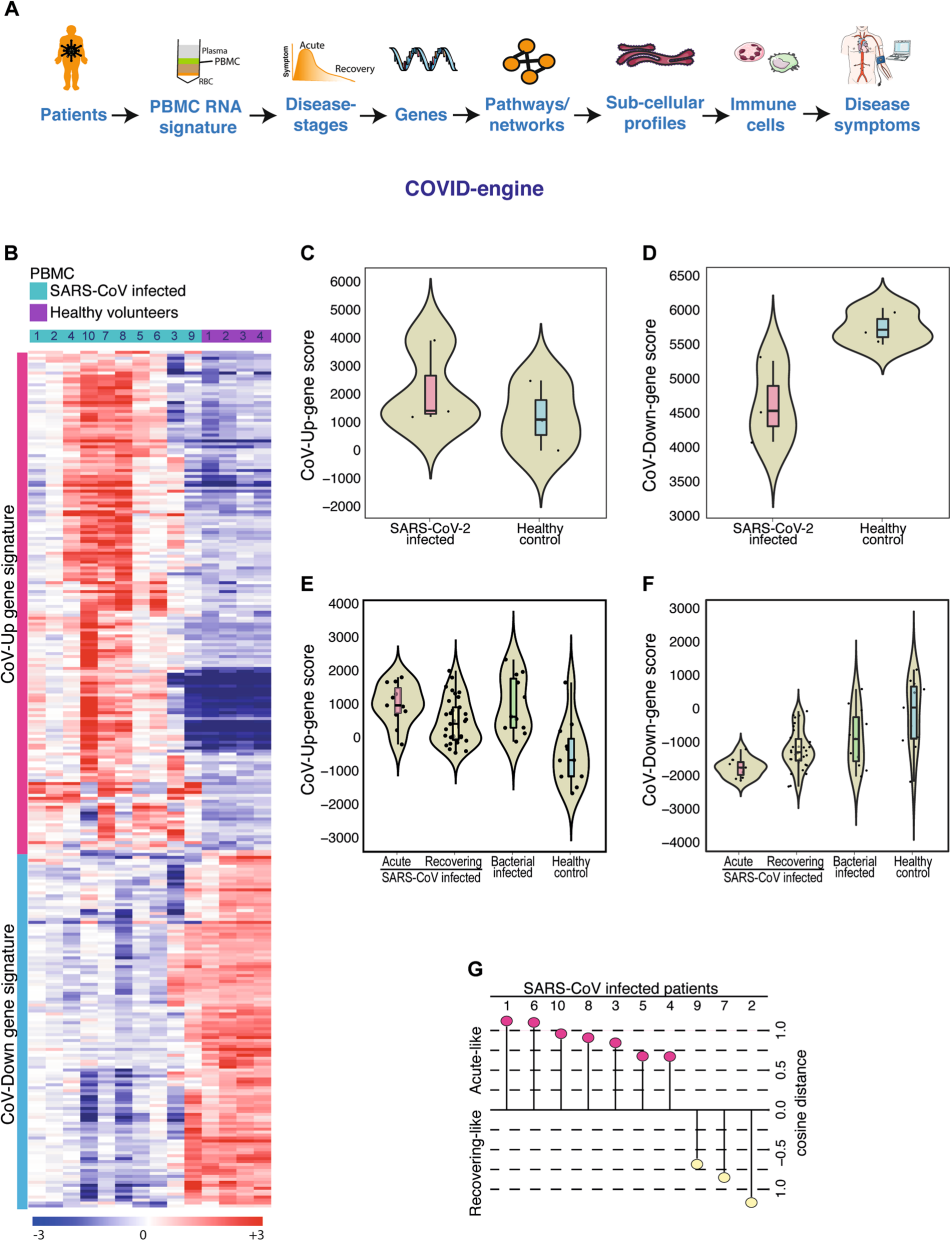
System-level analysis and PBMC-based gene signatures show association with SARS and COVID-19. **A** Schematic showing the identification of PBMC RNA gene signatures associated with disease staging, and hierarchical modeling of genes, pathways, networks, subcellular contents, cells, and disease symptoms using COVID-engine platform. This figure was prepared using Servier Medical Art (https://smart.servier.com) under a Creative Commons Attribution 3.0 Unported License (http://creativecommons.org/licenses/by/3.0/). **B** Heatmap showing 290 gene signature genes in 10 SARS patients and 4 healthy individuals. Both CoV-Up-gene signature (169 genes) and CoV-Down-gene signature (121 genes) are shown. **C**, **D**. CoV-Up-gene signature scores (**C**) and CoV-Down-gene signature scores (**D**) and their association with SARS-CoV-2-infected (COVID-19; *n* = 3) patients and healthy individuals (*n* = 3; datasets from Xiong et al.^17^). For **C** and **D** statistical significance was not considered due to low sample size. **E**, **F**. CoV-Up-gene signature scores (**E**) and CoV-Down-gene signature scores (**F**) and their association with acute and recovering SARS-CoV-infected patients (*n* = 25; 44 samples), bacteria-infected patients (*n* = 16), and healthy individuals (*n* = 9; datasets from Lee et al.^19^). Kruskal–Wallis statistical test with *p* < 0.001 for **E** and **F**. **G** Acute vs. recovering gene signature from Lee et al.^19^ predicted acute-like and recovering-like SARS-CoV-infected patients (*n* = 10) from Raghunathan et al.^16^ using NTP method and cosine distance measure.

In this study, we sought to understand and infer changes in coronavirus-infected patients at system levels through their peripheral blood mononuclear cells (PBMC) by performing comparisons with healthy volunteers by applying inferential statistics (IS). The inferences at the multiple levels of the system in patients provide an efficient way of understanding the heterogeneity and mechanism(s) of disease manifestation as a whole (Fig. 1A). These inferences can be derived systematically in a hierarchical fashion from the level of gene signatures to the whole organism, to study the pathophysiology of patients with coronavirus infection. Moreover, the patients’ response to insults from the virus along with other associated disease conditions can be studied.

## Results

### PBMC-based gene expression profiles identify distinct gene signatures in coronavirus-infected patients and healthy volunteers

To perform an integrative and systematic analysis of heterogeneous patients’ responses to the coronavirus infection, we used a limited RNA transcriptome data from PBMC of SARS-CoV patients (*n* = 10) and healthy volunteers (*n* = 4) from a published study^16^ (training data). We identified PBMC genes differentially expressed between patients and healthy volunteers by applying our in-house developed IS pipeline (“COVID-engine”; see “Methods“). We broadly identified 290 differentially expressed genes in patients and healthy volunteers using a supervised Statistical Analysis of Microarrays (SAM) approach (Fig. 1B, Supplementary Fig. 1 and Supplementary Tables 1A, B). Among these 290 genes, 169 (dubbed as CoV-Up-gene signature) were highly expressed, and 121 highly reduced in patients compared to healthy volunteers (dubbed CoV-Down-gene signature; Fig. 1B and Supplementary Table 1B).

We further explored our gene signatures in SARS-CoV-2-infected patients using a very limited sample size of three COVID-19 patients’ and three healthy volunteers’ PBMC from another published study by Xiong et al.^17^. Irrespective of different diseases (although related) and platforms, we observed that our CoV-Up-gene signature from SARS-CoV was higher in COVID-19 patients’ PBMC than in the healthy volunteers (see “Methods”). In contrast, CoV-Down-gene signature was higher in healthy volunteers and lower in COVID-19 patients. This result suggests that our gene signatures from SARS-CoV may be applicable to COVID-19 patients (Fig. 1C, D and Supplementary Table 1C).

When our CoV-Up-gene signature was analyzed using PBMC samples^18,19^ (*n* = 213) from patients infected with bacteria and influenza, we observed a broadly similar pattern (Fig. 1E, Supplementary Fig. 2A and Supplementary Table 1D, E). The reciprocal analyses with CoV-Down-gene signature was higher in PBMC from healthy volunteers than from SARS-CoV and other microbe-infected patients (Fig. 1F, Supplementary Fig. 2B and Supplementary Table 1D, E). Again, we performed enrichment analysis (hypergeometric test) using MSigDB’s C7 immune signature^20^ and found that 43% (60 out of 138 genes) of the signatures that were derived from PBMC (mostly associated with specific diseases) were significantly (false discovery rate (FDR) < 0.2) enriched for our CoV-Up-gene signature (Supplementary Fig. 3). Overall, the results suggest that CoV-Up-gene signature represents primarily diseased PBMC.

### PBMC gene signatures may distinguish disease progression—acute-like vs. recovering-like coronavirus-infected patients

Next, we sought to assess the potential of our CoV-Up-gene signature to stratify patients into those at different progression stages of the disease (progression): acute vs. recovering. For this, we used PBMC transcriptome data from a limited size of 44 samples from the longitudinal collection over the disease course from a published study (validation data; ref. ^19^). Lee et al. defined acute samples (*n* = 25) as those that tested positive (using blood) for SARS-CoV during hospitalization or within 10 days of onset of the disease in patients. The samples derived from acute patients were also correlated with disease severity including an increased clinical pulmonary infection score^19,21^. The remaining samples were labeled as recovering samples (*n* = 19). Hence, acute vs. recovering samples refer to different stages of the disease that can occur in the same patient as specific samples were collected from the same individual during hospitalization.

Interestingly, disease phase appeared to be associated with our identified CoV expression signatures in samples from Lee et al.^19^ with recovering patients showing an intermediate signature between acute phase and healthy donors (Fig. 1E). We then applied a gene signature (39 genes with known gene symbols) for distinguishing acute from recovering patients identified by Lee et al.^19^ back to our training data^16^ using NTP analysis (see “Methods”)^22^. This resulted in 7 of the 10 patient samples showing maximal similarity to acute phase (termed acute-like patients), while 3 samples scored similar to recovering patients (termed recovering-like patients; measured as signature-based cosine distance; see “Methods”; Fig. 1G and Supplementary Table 1F). Our results therefore suggest that CoV signatures may distinguish different disease stages.

### Gene expression of key cytokines and other genes represented in PBMCs in acute-like vs. recovering-like coronavirus patients

**Fig. 2:**
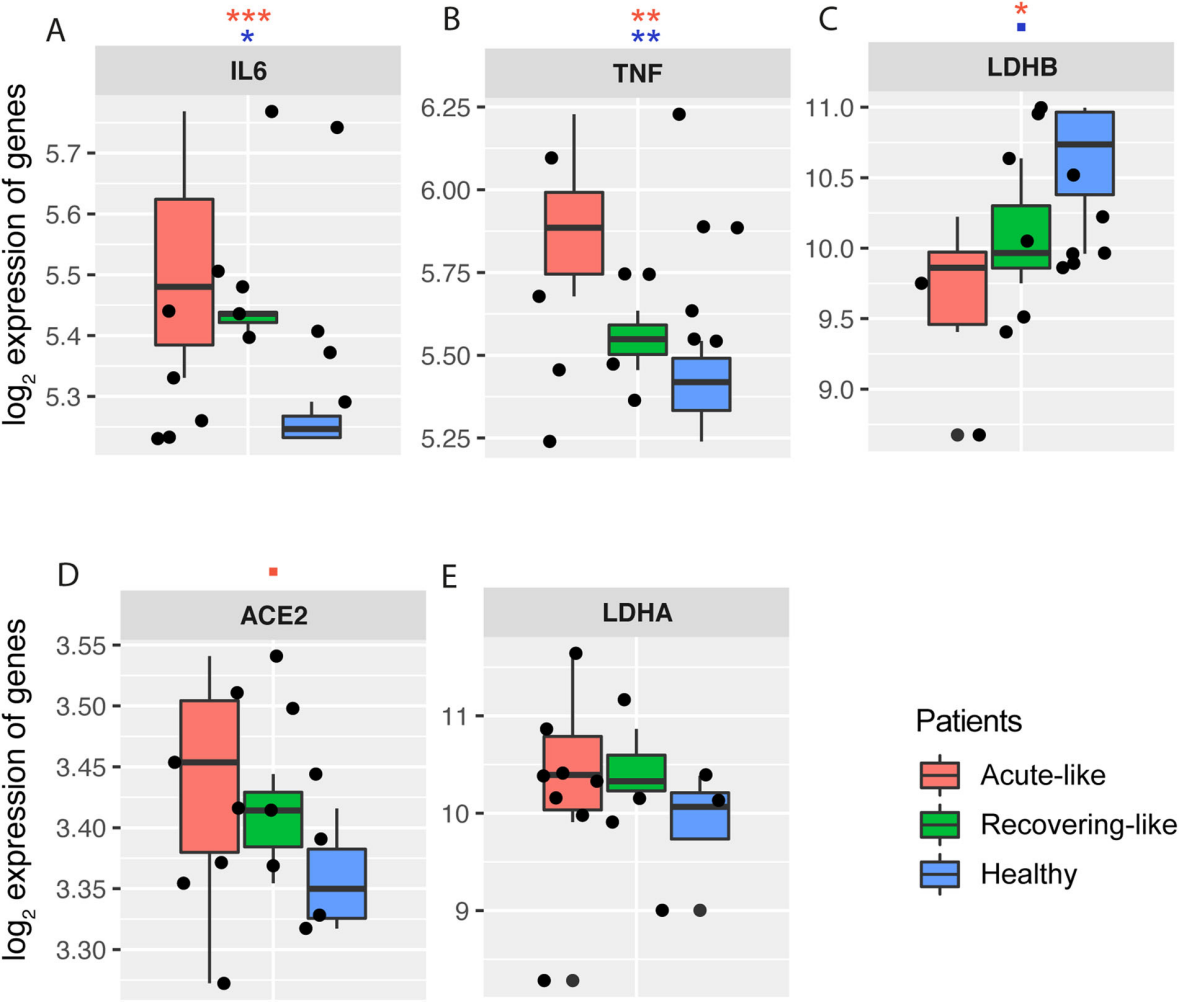
Significantly represented key genes at different progression stages of coronavirus infection. **A–E** Expression levels of genes: *IL6* (**A**), *TNF* (**B**), *LDHB* (**C**), *ACE2* (**D**), and *LDHA* (**E**) in acute-like and recovering-like SARS patients and healthy individuals. Blue asterisk (*) represents Kruskal–Wallis nominal *p* value across all three groups and red asterisk (*) represent Welch two-sample *t-*test *p* value between patients and healthy individuals. *p < 0.05; **p < 0.001; ***p < 0.0001 and ^■^*p* < 0.08. Multiple testing was not done due to low sample size.

We next analyzed the expression patterns of genes associated with coronavirus infection in PBMCs in predicted acute-like and recovering-like using SARS-CoV-infected patients and healthy volunteers (from training data). We compared the expression levels of cytokines namely *IL6* and *TNF* and other genes such as *ACE2* and lactate dehydrogenases in PBMC from infected patients and healthy individuals, which are known to be expressed in circulating monocytes and macrophages after viral infection^23,24^. Although increased expression level of *IL6* in acute-like patients compared to healthy individuals was observed, there was no significant difference between acute-like and recovering-like patients (Fig. 2A). Interestingly, *TNF* was highly expressed in acute-like patients compared to recovering-like patients and healthy individuals (Fig. 2B). Besides, analysis of *ACE2* and subunits of lactate dehydrogenase (*LDHA* and *LDHB*; associated with hypoxia) genes showed *LDHB* was highly expressed in healthy individuals compared to coronavirus-infected patients, and inverse trends were observed for *ACE2* and *LDHA* (Fig. 2C, E). Among these five genes, *TNF* was the only gene that showed differential expression between acute-like and recovering-like patients. The results show increased expression of these key coronavirus infection associated genes in patient PBMC samples.

**Fig. 3:**
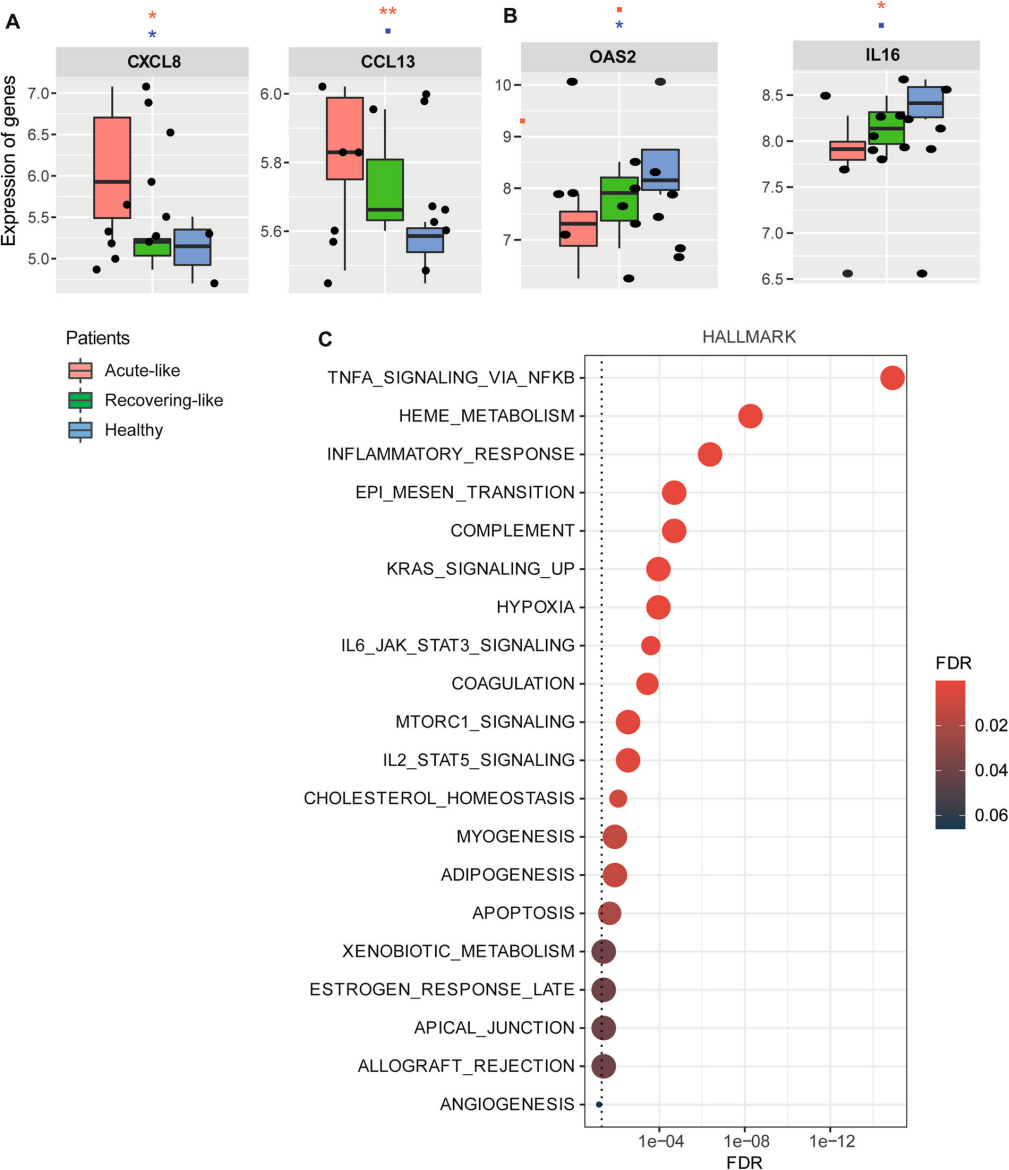
Highly represented signaling pathways/processes in coronavirus-infected patients. **A**, **B** Expression levels of genes related to immune cell chemoattractants *CXCL8* and *CCL13* (**A**), genes involved in T cells and suppression of viral replication *OAS2* and *IL16* (**B**) in acute-like and recovering-like coronavirus-infected patients and healthy individuals. Blue asterisk (*) represents Kruskal–Wallis nominal *p* value across all three groups and red asterisk (*) represent Welch two-sample *t-*test *p* value between patients and healthy individuals. *p < 0.05; **p < 0.001; ***p < 0.0001 and ^■^*p* < 0.08. Multiple testing was not done due to low sample size. **C** Enrichment statistical analysis (hypergeometric test using hypeR^57^) using CoV-Up-gene signature and pathways/processes based on gene sets from MSigDB’s HALLMARKS database^20^.

We further examined multiple other candidate genes that act as chemoattractants to monocytes and macrophages, specifically those that interfere with innate and adaptive immunity and viral replication^24^. Among those genes, we observed *CXCL8* (*IL8*) and *CCL13*, which are associated with chemoattraction of neutrophils/macrophages (innate immunity), to be highly expressed in acute-like patients, compared to recovering-like patients and healthy individuals (Fig. 3A). On the other hand, *OAS2* and *IL16* associated with T cells (adaptive immunity) and inhibition of viral replication were highly expressed in recovering-like patients and healthy individuals (Fig. 3B). These results suggest that PBMC from potential acute-like patients may be associated with the activity of innate immunity, whereas PBMC from recovering-like patients may be associated with an adaptive immune profile.

### Enrichment of TNF-alpha, *IL6*, and hypoxia-related pathways in PBMC of coronavirus patients

Based on the expression levels of key genes, including *TNF*, we next set out to explore the functional implications of the CoV-Up-gene signature. To do so, we performed enrichment analysis using the genes in CoV-Up-gene signature and MSigDB’s hallmark gene set database^20^ (Fig. 3C). This revealed multiple highly ranked pathways involved in cytokine storm and acute infection including TNF signaling, IL6 (IL6-JAK-STAT3) and IL2 (IL2-STAT5) signaling, inflammatory response, and KRAS/MTOR and late responses to estrogen pathways (Fig. 3C and Supplementary Table 2A). This is consistent with clinical manifestations including observations of high IL6 levels in COVID-19 patients^25^.

Interestingly, outside of the inflammatory response, multiple pathways related to hypoxia, angiogenesis, and oxygen transport (heme/iron) were also implicated, consistent with the oxygen limitation^26^ experienced during coronavirus infection (Fig. 3C). Of particular interest was the enrichment of complement and coagulation pathways which may explain the high hypercoagulability observed in COVID-19 patients^27^ and may represent one of the key pathological mechanisms of the virus. Enrichment of the apoptotic pathway may have relevance to cell death of lymphocytes (Fig. 3C) as suggested elsewhere^17^. These enriched pathways and processes may be linked together and may represent an association with COVID-19 infection in these patients.

### Potential role for a network of related pathways representing cytokine storm and innate immune changes in PBMC of coronavirus-infected patients

**Fig. 4:**
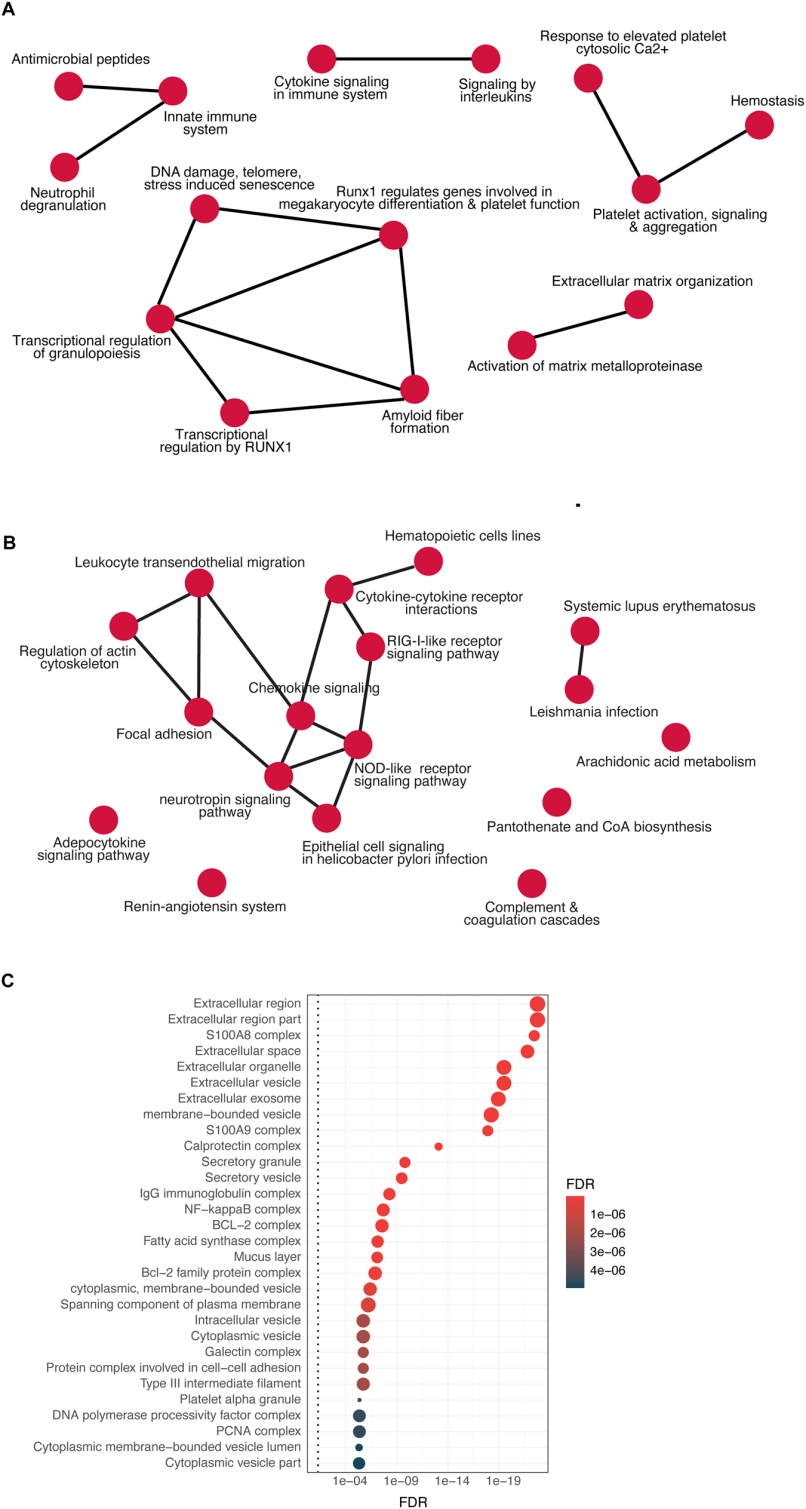
Highly represented related networks of molecular, cellular, and development pathways show cytokine (storm) network and innate immunity in coronavirus-infected patients. **A** REACTOME^28^ database-based connection of different but related pathways that were enriched (hypergeometric test of FDR < 0.2 and overlap a similar index of 0.5 using hypeR^57^) in infected patients using network plots. **B** KEGG^60^ pathways-based network showing enrichment (hypergeometric test of FDR < 0.2 and overlap a similar index of 0.25 using hypeR^57^) of different diseases and infection related pathways. For **A** and **B**, nodes and edges are of same size and length, respectively. **C** Enrichment statistical analysis (hypergeometric test of FDR < 0.2 using hypeR^57^) using CoV-Up-gene signature and pathways/processes based on gene sets from COMPARTMENTS database^61^.

Given that different pathways were enriched in infected patients, next, we interrogated how these pathways are linked together to convey a network of processes or changes at the cellular level. Hence, we used the REACTOME pathway database^28^ to connect different but related pathways that were enriched in infected patients using network analysis. Two evident and distinct networks were significantly enriched in CoV-Up-gene signature: (a) interleukins and cytokine signaling (potentially representing cytokine storm) and (b) neutrophils and innate immunity (Fig. 4A and Supplementary Table 2B). Nevertheless, we observed an increased enrichment of a unique network linking granulopoiesis, megakaryocyte differentiation, and platelet activation (Fig. 4A). This may be linked to coagulation system that can activate the innate immune system (e.g. monocytes/macrophages) to produce TNF^29^. Nevertheless, this requires further understanding. These may suggest innate immune system activation with potential cytokine storm in coronavirus patients.

Kyoto Encyclopedia of Genes and Genomes (KEGG)-based extensive analysis of network of molecular pathways suggested further understanding of functions and utilities at the levels of cells and organisms. Our KEGG analysis with CoV-Up-gene signature overlapped with different infections, including *Helicobacter pylori* and leishmania (Fig. 4B and Supplementary Table 2C). This suggests that coronavirus infection may share disease conditions with these infections. Interestingly, we also noted enrichment of systemic lupus erythematosus (SLE)-related genes, which is a chronic disease associated with inflammation in connective tissues and affects multiple organs, including the blood-forming system^30,31^. This suggests that coronavirus infection and SLE may be related to each other with respect to disease symptoms. The prevalence and risk factors of severe COVID-19 in SLE patients remain unclear^30,31^. The understanding at the systemic level and additional clinical reports are needed to know whether certain COVID-19 disease conditions may be associated with SLE. A more relevant and well-known individual pathway associated with this disease is the renin–angiotensin system associated with *ACE2* function (Fig. 4B). There is also an enrichment of RIG-I-like receptor signaling pathway representing potential anti-viral event through pathogen-associated molecular patterns^32^. Multiple chemokine/cytokine and metabolism pathways were enriched as a part of the CoV-Up-gene signature in the KEGG database (Fig. 4B).

### Changes in subcellular regulatory networks associated with PBMC of coronavirus-infected patients

While these genes to network processes provide information related to coronavirus infection, we were interested in investigating the next level in the hierarchy and the potential subcellular interaction networks that may inform viral interaction within host cells. Interestingly, cell–cell adhesion processes, secretory granules, vesicles, and exosomes spanning plasma membrane and lipid complexes and cytoplasm were enriched in CoV-Up-gene signature (Fig. 4C), suggesting that this may indicate the viral infection of monocytes. Furthermore, these were related to the enrichment of fatty acid synthase complex that is known to be involved in the plasma membrane and vesicle formation^33^ (Fig. 4C and Supplementary Table 2D). The data also suggests potential interaction of the virus with host immune cells through cell–cell adhesion processes, which requires further understanding.

Nonetheless, the host-specific subcellular changes in PBMC are also evident from this analysis. An increased replication and proliferation of potential host cells, mainly involving the innate immune system, may be evident based on the enrichment of genes associated with DNA polymerase processivity factor and proliferating cell nuclear antigen complex. Also, the production of immunoglobin complexes along with NFkB complex was higher in the patient gene signature, again, representing potentially increased immune responses. At the same time, the host’s potential responses to death signals associated with BCL2 complex are also enriched in this analysis. Again, this potentially represents lymphocyte-related apoptosis in connection with an enriched apoptotic pathway in Fig. 3C. Neutrophil-specific S100A8/A9 complexes are also enriched (Fig. 4C). Overall, these results suggest potential PBMC-based subcellular level changes associated with the viral integration in immune cells and associated pathophysiology.

### Recovery from coronavirus infection is potentially associated with increased cytolytic activity and IFN-*γ* but not increased B cell levels

**Fig. 5:**
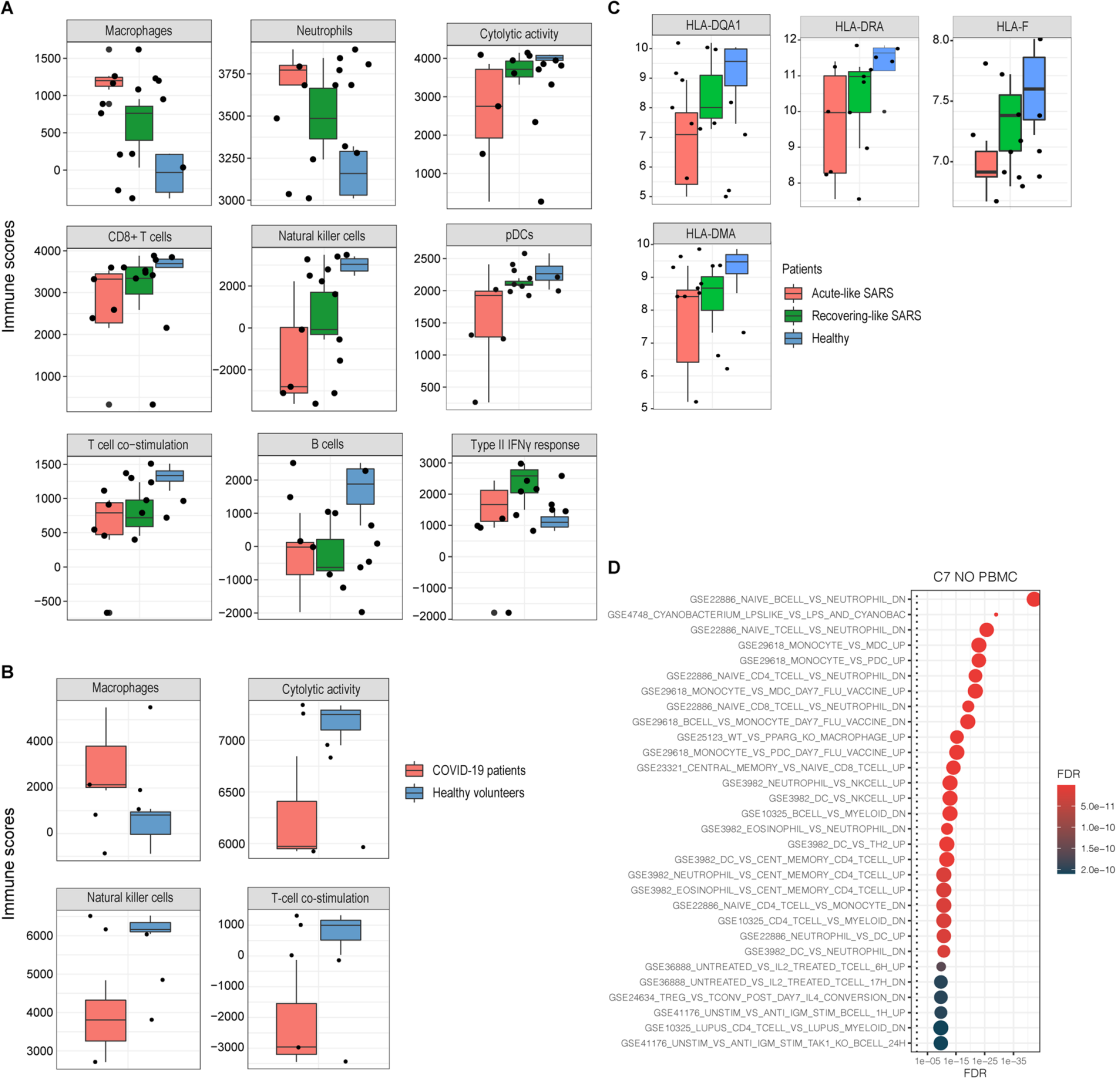
Immune cells, their activities and HLA types in progression stage-specific coronavirus-infected patients. **A**, **B** Immune cell scores using ssGSEA analysis and specific immune signature from Rooney et al. representing specific immune cells and their activities in acute-like (*n* = 7), and recovering-like (*n* = 3) SARS patients and control healthy individuals (*n* = 4) (**A**), and COVID-19 patients and control healthy individuals (**B**). FDR for **A** and **B** are <0.2. **C** HLA profiles in acute-like, and recovering-like SARS patients and control healthy individuals. Kruskal–Wallis statistical nominal *p* values for *HLA-DQA1* and *HLA-DOB* are <0.1 and *HLA-F* is <0.05. Multiple testing correction was not performed due to small sample size for **C** (the intention of the study is to infer from publicly available limited data). **D**. Enrichment analysis (hypergeometric test using hypeR^57^) of CoV-Up-gene signature using MSigDB’s C7 immune gensets^20^.

In order to gain insight into the cellular dynamics of the SARS-CoV-2 immune response, we calculated immune signature scores based on the gene markers from Rooney et al.^34^ (see “Methods”). In this case, we separated the coronavirus patient samples into those with acute-like or recovering-like disease and compared these with healthy control samples. As expected, we observed that the innate immune system involving macrophages and neutrophils were highly active in the acute-like patients, suggesting that they may be the first to encounter the coronavirus, with these decreasing in recovering patients (Fig. 5A and Supplementary Table 2E).

Perhaps most interestingly, a significant (FDR < 0.2) increase in natural killer (NK) cells, cytolytic activity, and plasmacytoid dendritic cells (pDCs) in recovering-like patients compared to acute-like patients was observed (Fig. 5A). It is noteworthy that the absolute levels of *CD8*^+^ T cells and co-stimulating helper T cells are not different between recovering-like and acute-like patients (Fig. 5A). This result suggests that the *CD8*^+^ T cells are potentially activated (cytolytic) in the recovering patients. Certain results from SARS patients were assessed using PBMC from limited number of COVID-19 samples (*n* = 3; FDR < 0.2; Fig. 5B and Supplementary Table 2F). No difference in B cells between both types of patients and healthy individuals was found. Intriguingly, higher level of expression of interferon (IFN)-γ type-II gene was found in the recovering-like patients and lower levels in both acute-like patients and healthy volunteers (Fig. 5A). These results suggest that T cell responses may be pivotal in successful response to coronavirus infection, consistent with the recent study by Grifoni et al.^35^ which found SARS-CoV-2 reactive T cells in 70% of convalescent COVID-19 patients.

Next, we examined the differential expression of major histocompatibility complex (MHC) class-I and class-II HLA that may reflect antigen presentation to and/or activation of *CD4*^+^/*CD8*^+^ T cells, and whose levels are increased by IFN-γ (Fig. 5C). Among the MHC class-II HLA genes, two of them were lower in acute-like patients compared to recovering-like and/or healthy individuals (borderline significance with nominal *p* < 0.1 due to low sample size). While most of the MHC class-II HLA genes showed no difference in expression levels between the acute-like and recovering-like patients, *HLA-DQA1* showed an increasing trend in the recovering-like patients towards the healthy individuals and low level in acute-like patients. Similarly, MHC class-I HLA gene, *HLA-F* showed a trend akin to *HLA-DQA1* (Fig. 5C and *p* < 0.05). On the other hand, there was no change in the antigen processing machinery (data not shown). However, all these speculations from limited data warrant further validation, and their functional immunological significance is currently unclear.

**Fig. 6:**
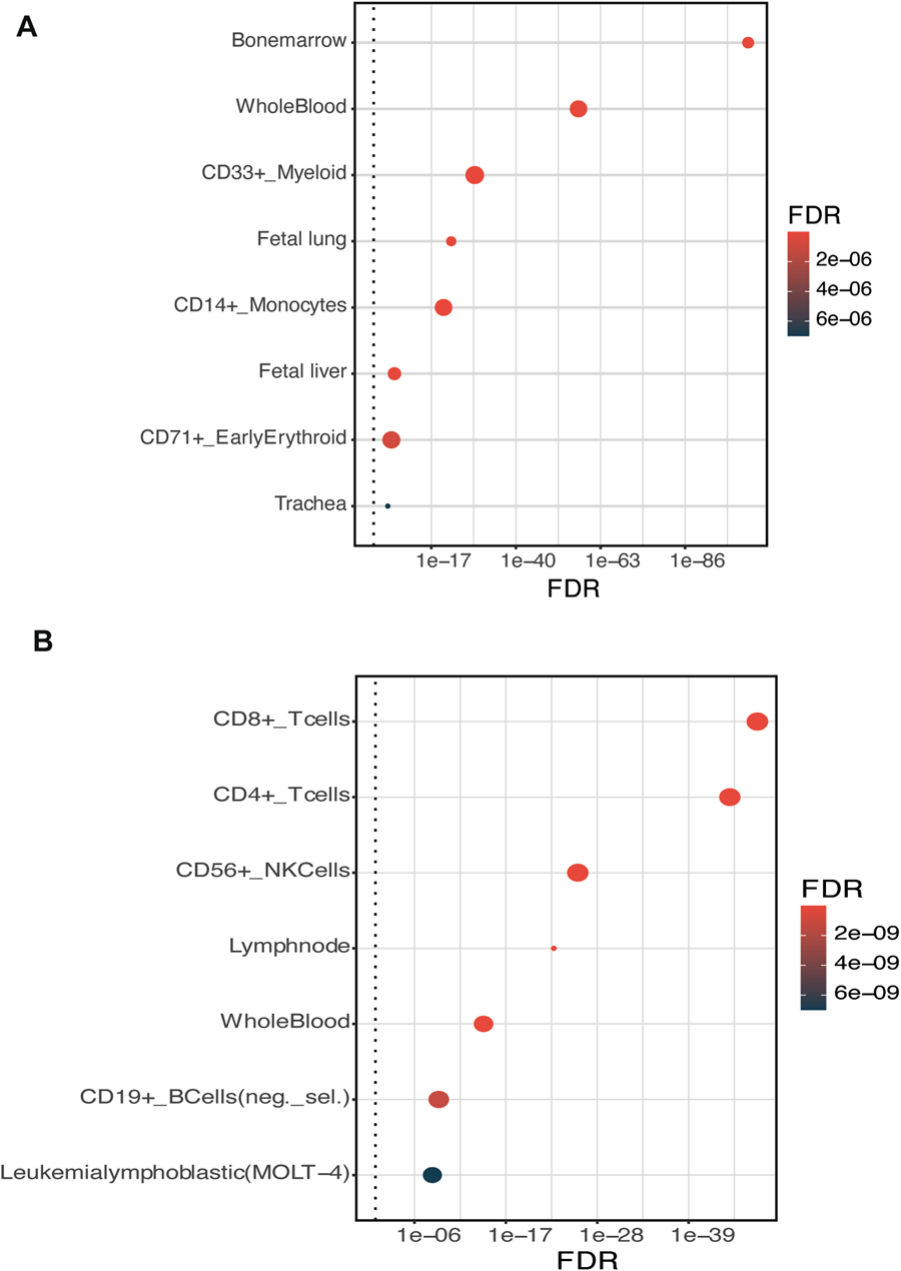
PBMC-based gene signatures show association with subset of immune cells in coronavirus-infected patients. **A**, **B** Enrichment (hypergeometric test using hypeR^57^) of subsets of immune cell genes in multiple BioGPS—gene portal system^36^ using CoV-Up-gene signature (**A**) and CoV-Down-gene signature (**B**).

To confirm these analyses of immunologic composition, we performed two additional independent analyses. Hypergeometric test-based enrichment analysis of MSigDB’s C7 immune signature^20^ showed similar conclusions that innate immunity and myeloid (neutrophils and macrophages) cells are upregulated in coronavirus patients (in general), whereas adaptive immunity is downregulated in these patients (Fig. 5D and Supplementary Table 2G). Similarly, analysis using the BioGPS database—gene sets^36^ demonstrated an increased enrichment of CD33^+^ myeloid and *CD14*^+^ monocytes associated with upregulated genes in our CoV expression signature (Fig. 6A and Supplementary Table 2H). In contrast, *CD8*^+^ and *CD4*^+^ T cells showed up along with enrichment of *CD56*^+^ NK cells and *CD19*^+^ B in the CoV-Down-gene signature (Fig. 6B and Supplementary Table 2I). Compared to healthy individuals, these cells were under-represented in CoV-Up-gene signature or lower in coronavirus-infected patients (Fig. 6B).

### Coronavirus-infected patients’ PBMC reveals genes that overlap with known diseases and disease conditions

**Fig. 7:**
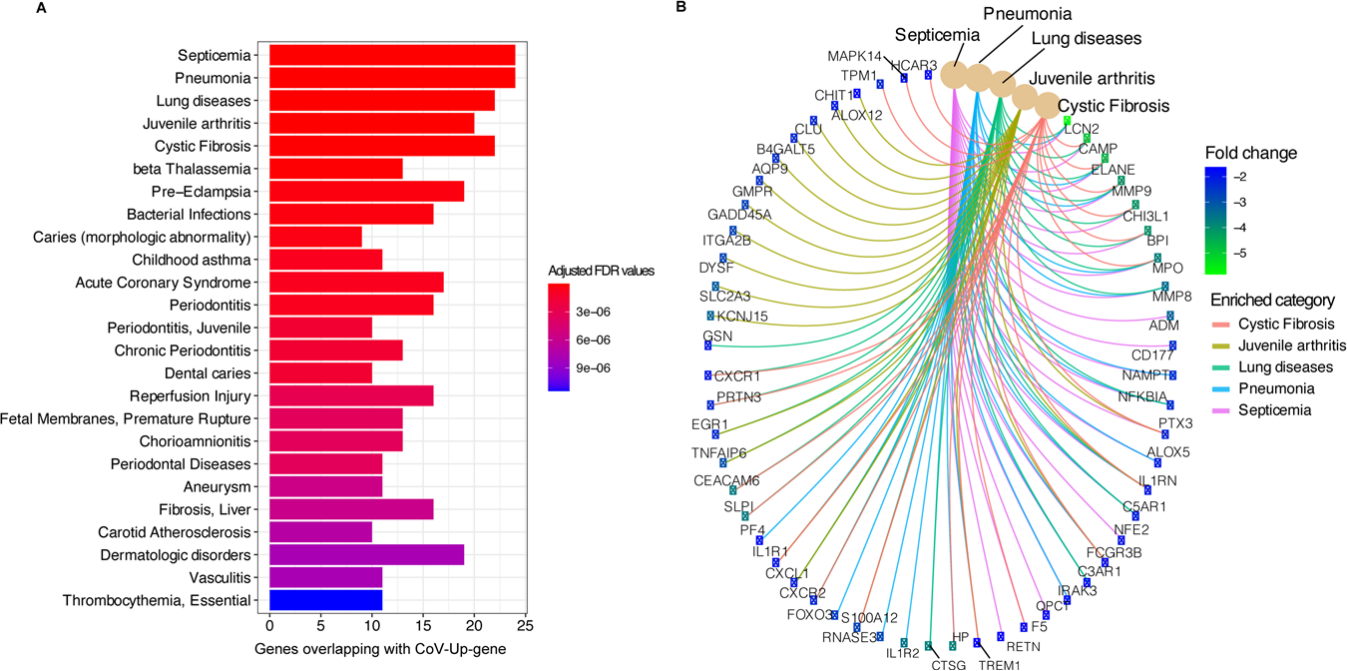
PBMC-based gene signature identifies links to known and novel diseases and disease conditions in coronavirus-infected patients. **A**, **B** Enrichment analysis (hypergeometric test using hypeR^57^) of disease-based gene sets from DisGeNET^62^ (**A**). Top five diseases and associated PBMC genes (**B**).

Based on information in Supplementary Fig. 3, we reasoned that the disease conditions from coronavirus infection may be similar to other immune-related diseases. To perform this, we applied enrichment analysis to study the overlap of genes between CoV-Up-gene signature and other diseases and disease conditions. We found that CoV-Up-gene signature was enriched for various immune-related diseases, including septicemia, pneumonia, lung disease, arthritis, cystic fibrosis, thalassemia, pre-eclampsia, bacterial infections, asthma, acute coronary syndrome, and others (Fig. 7A and Supplementary Table 2J). The overlap of CoV-Up-gene signature and those genes from selected diseases—septicemia, pneumonia, lung diseases, arthritis, and cystic fibrosis—are shown in Fig. 7B. These results suggest that the diseases and disease conditions due to coronavirus may be complex and highly variable and may affect differently in patients with pre-existing disease conditions as recently reported^7^, which warrants further systematic investigation.

## Discussion

The clinical course of COVID-19 patients remains enigmatic, and no treatment options exist with proven efficacy^37^. The variety of clinical presentations of this disease has alarmed healthcare providers across the globe. The rampant spread of COVID-19 during asymptomatic stage is attributed to the high SARS-CoV-2 viral shedding in the upper respiratory tract^38^. We reasoned that the blood, in real-time, may reflect changes occurring in immune and other cells and potentially infected tissues in PBMC, thereby acting as a potential remote biosensor of highly complex system-wide changes. There is no systematic study performed to our knowledge that attempts to use PBMC samples to understand the system-wide changes along with the disease symptoms in COVID-19 patients. This type of study will have the strength to distinguish systemic changes during acute and recovering stages of the patient’s infection. This proof-of-concept study, in the future with further refinement and extensive validation, may support the development of personalized prognostic biomarkers (not the focus of the current study) and may provide the opportunity to save patients who are most likely to die of the disease.

In this proof-of-concept study, we performed a comprehensive analysis using publicly available blood cell RNA profiles from SARS and COVID-19 patients and cross-validated with patients with other infections or healthy individuals. Using IS approaches in our COVID-engine platform, we were able to develop a blood cell RNA profile-based gene signatures that are differentially expressed at different stages of infection (acute vs. recovering). In addition, our COVID-engine platform provided hierarchical and comprehensive analysis describing infection-associated changes in genes, pathways, networks, subcellular components, and cells, covering almost the whole system. This “integrated” analysis can help understand which other disease-related symptoms could manifest in COVID-19 patients.

Our results, using limited coronavirus-infected patient samples, represent that the innate immune system associated with increased neutrophils, macrophages, and monocytes with potential cytokine storm (including the expression of *IL6*, *TNF*, *IL8* (*CXCL8*) and *CCL13*) is high in CoV-Up-gene signature and specifically in acute-like patients. Macrophages and monocytes are known to serve as factories for viral replication in other disease conditions^24^. These changes in immune cells may also be connected to increased neutrophil counts in these patients^39^. The cytokine storm-related to innate immune changes may be linked to changes in angiogenesis and coagulation, suggesting a potential relationship between inflammation, thromboembolism^40^, and coagulation^41^. While there is no change in overall *CD8*^+^ T cell population between patients in their acute-like vs. recovering-like stages (that may, however, attribute to the sample size), the change in cytolytic activity, pDCs, and NK cells suggests that the adaptive immunity is a late event represented in patients recuperating from this disease^42^. Congruently, this is associated with lower innate immunity in recovering-like patients than acute-like patients and associated with increased expression of anti-viral genes *OAS2* and *IL16*.

Similarly, the higher expression levels of MHC Class-I *HLA-F* gene, which is known to be associated with the interaction between CD4 T cells and NK cells to inactivate human immunodeficiency virus (HIV)^43^, in recovering-like patients suggests the anti-viral effect in these individuals. Remarkably, the supposedly anti-viral CD4 T and NK cells, along with B cells, are low in coronavirus patients and are associated with low *HLA-DR-*expressing monocytes in these patients with severe respiratory failure^25,44,45^. This report corroborates with our results that *HLA-DRA* gene and all the above three cell types are low in acute-like patients. Specifically, B cell-based adaptive immunity seems to vary among patients and mostly low in coronavirus patients with severe respiratory failure^25,46^. Our data suggest that this may impact the development of effective vaccines for this infection. In addition, CoV-Up-gene signature was high in methicillin-resistant *Staphylococcus aureus* (Supplementary Fig. 2), which, similar to coronavirus, colonizes upper respiratory track and causes pneumonia. It is interesting to note that there is no vaccine for *S. aureus* infection^47^. While there are more changes in T cells than B cells, it may be interesting to consider T cell therapy for COVID-19 patients^48^. In our study, there are disease links and potential comorbidities (Fig. 7) that has evidence from recent reports^7^. Further refinement of our current COVID-engine platform may help to identify hidden/silent pre-existing symptoms and develop an effective personalized COVID-19 treatment strategy using PBMC.

There are a number of questions that arose from this study that could be of relevance in tackling the current pandemic. How does coronavirus infection downregulate the adaptive immune system? Is the dysregulation of the immune system described causally linked with clinical outcomes? Does the dysregulated immune system alert the body to respond, and how? The dysregulated immune system could alert the host response to produce an active adaptive immune response, which typically takes 10–14 days. Our findings may suggest that individuals with activated, appropriate immune responses, especially with increased INF-γ type-II responses and cytolytic activity, which may also serve as biomarkers with further validation, maybe on their way to recovery from symptoms. Moreover, those patients’ incapable of such progression may have multiorgan failures that may be represented in our data.

Although our study may be timely for the current pandemic, there are limitations. We have performed analysis using publicly available small number of coronavirus-based training (*n* = 10) and test (*n* = 47) samples from less annotated datasets with limited clinical data, which may be appreciated provided the current global lock-down scenario. Also, the acute-like and recovering-like patients may overlap with the symptomatic and asymptomatic patients, respectively, described in the original publication from where the training dataset was derived^16^. Further validation was curtailed due to lack of associated clinical data, which is difficult to obtain in the current scenario.

**Fig. 8:**
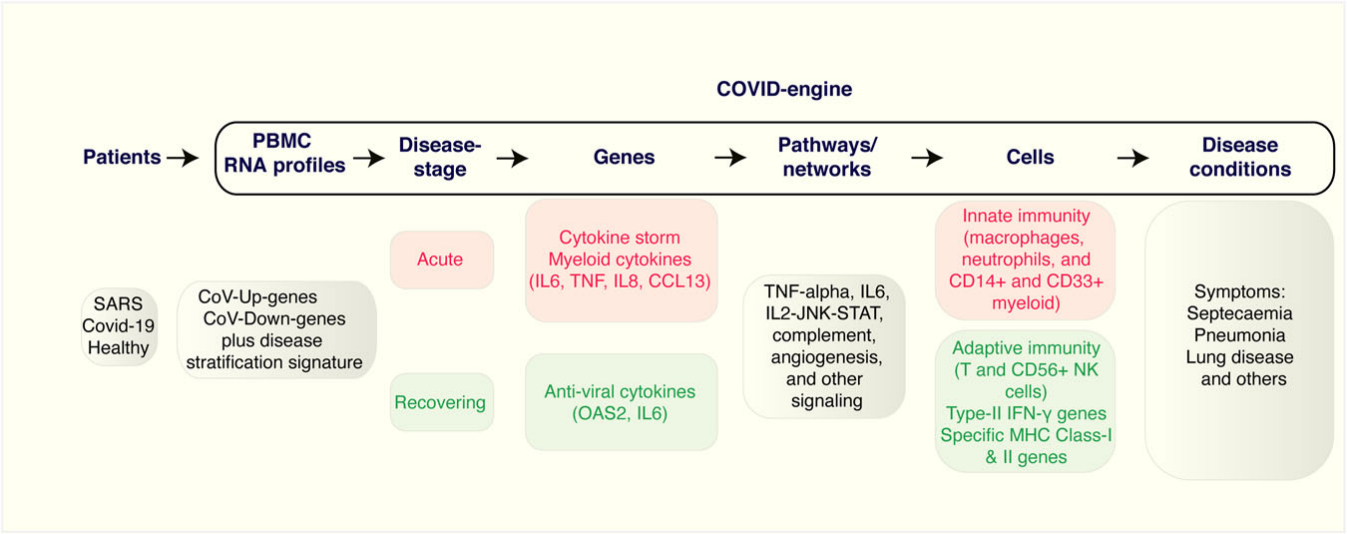
COVID-engine workflow. Schematic summarizing the gene signature and their relevance at systems level to disease progression stages, and disease conditions for personalized COVID-19 medicine.

In conclusion, PBMC has information related to infection status, immune states, disease progression, severity, and disease conditions that are likely going to be manifested due to coronavirus infection and COVID-19 disease (Fig. 8).

## Methods

### Data sources

PBMC transcriptomes from SARS, COVID-19, and other patients were obtained from published studies^16–19^ with GEO Omnibus identifiers—GSE1739 and GSE6269, and normalized data directly from the original publications. Raw.CEL files from Affymetrix Human HG-Focus Target Array for GSE1739 were obtained from the authors of the original publication^16^ and robust microarray analysis-based normalization was performed using R-based Bioconductor package—*affy*^49^. GEOquery R package^50^ was used to obtained gene expression and/or phenotypic data from GEO Omnibus for GSE6269.

### Overall analysis strategy

COVID-engine refers to a pipeline of different IS methods described below.

#### Differential PBMC gene expression analysis and validation

PBMC-based differential gene signatures between healthy volunteers and coronavirus-infected patients were selected by performing supervised SAM^51^ using R-based *siggenes* package^52^. Differentially expressed genes with FDR < 0.05 were chosen as described in our previous publications^53–55^. Gene scores for signatures up or downregulated in coronavirus-infected patients in other validation datasets were derived using ssGSEA^20^ using R scripts from GenePattern platform^56^.

#### Enrichment and other IS analysis for systems level understanding of the disease

Briefly, the differentially expressed gene expression signatures from PBMC of patients infected with coronavirus were used to query multiple databases for meta-signatures such as pathways, mechanistic processes, and their associated networks that are connected with different disease manifestations by enrichment analysis-based IS. These gene signatures and meta-signatures were further systematically linked to wire the pathophysiology in patients, again, in a hierarchical fashion, from cells to whole organism level (Fig. 1A).

Hypergeometric gene enrichment analysis for most of the studies were performed using R-based hypeR tool^57^. Disease conditions-based enrichment analysis was performed using R-based Dose package^58^. Nearest Template Prediction^22^ using R-based tool from GenePattern platform^56^ was used to derive distance between two signatures—acute-like vs. recovering-like patients. Different gene set databases were from downloaded from EnrichR^59^ and MSigDB^20^. Immune gene sets were from Rooney et al.^34^. Additional gene sets and databases used in this study are: REACTOME^28^, KEGG^60^, COMPARTMENTS database^61^, BioGPS—gene portal system^36^ and DisGeNE^62^.

Detailed information regarding intermediate data from COVID-engine are provided in Supplementary Tables 1 and 2. The number of samples used in this exploratory study was determined by public availability of transcriptomic datasets and for the need of the hour. Kruskal–Wallis and *t-*test statistical analysis were performed where appropriate.

## Supplementary information

Supplementary Information

Supplementary Figure 1

Supplementary Figure 2

Supplementary Figure 3

Supplementary Table 1

Supplementary Table 2

## Data Availability

SARS, COVID-19, and other samples were obtained from published studies^16–19^ with GEO Omnibus identifiers—GSE1739 and GSE6269, and data directly from the original publications.

## Code availability

All R scripts for COVID-engine can be found at https://github.com/syspremed/COVID-engine.

## References

[1] World Health Organization, Coronavirus disease 2019 (COVID-19) Weekly epidemiological update. (1 December 2020); https://www.who.int/publications/m/item/weekly-epidemiological-update---1-december-2020.

[2] Wong JEL, Leo YS, Tan CC (2020). COVID-19 in Singapore—current experience: critical global issues that require attention and action. JAMA.

[3] Kindler E, Thiel V, Weber F (2016). Interaction of SARS and MERS coronaviruses with the antiviral interferon response. Adv. Virus Res.

[4] Shokri S, Mahmoudvand S, Taherkhani R, Farshadpour F (2019). Modulation of the immune response by Middle East respiratory syndrome coronavirus. J. Cell Physiol..

[5] Kikkert M (2020). Innate immune evasion by human respiratory RNA viruses. J. Innate Immun..

[6] Swerdlow DL, Finelli L (2020). Preparation for possible sustained transmission of 2019 novel coronavirus: lessons from previous epidemics. JAMA.

[7] Wadman M, Couzin-Frankel J, Kaiser J, Matacic C (2020). How does coronavirus kill? Clinicians trace a ferocious rampage through the body, from brain to toes. Science.

[8] Phelan AL, Katz R, Gostin LO (2020). The novel coronavirus originating in Wuhan, China: challenges for global health governance. JAMA.

[9] Nishiura H (2020). Estimation of the asymptomatic ratio of novel coronavirus infections (COVID-19). Int. J. Infect. Dis..

[10] Ng OT (2020). SARS-CoV-2 infection among travelers returning from Wuhan, China. N. Engl. J. Med..

[11] Bai Y (2020). Presumed asymptomatic carrier transmission of COVID-19. JAMA.

[12] Wang, L., Gao, Y. H., Lou, L. L. & Zhang, G. J. The clinical dynamics of 18 cases of COVID-19 outside of Wuhan, China. *Eur. Respir. J.*, 10.1183/13993003.00398-2020 (2020). 10.1183/13993003.00398-2020 PMC7098482 32139464

[13] Chen D (2020). Recurrence of positive SARS-CoV-2 RNA in COVID-19: a case report. Int J. Infect. Dis..

[14] Shanmugaraj B, Malla A, Phoolcharoen W (2020). Emergence of novel coronavirus 2019-nCoV: need for rapid vaccine and biologics development. Pathogens.

[15] Pang, J. et al. Potential rapid diagnostics, vaccine and therapeutics for 2019 novel coronavirus (2019-nCoV): a systematic review. *J. Clin. Med.* **9**, 10.3390/jcm9030623 (2020). 10.3390/jcm9030623 PMC7141113 32110875

[16] Reghunathan R (2005). Expression profile of immune response genes in patients with severe acute respiratory syndrome. BMC Immunol..

[17] Xiong Y (2020). Transcriptomic characteristics of bronchoalveolar lavage fluid and peripheral blood mononuclear cells in COVID-19 patients. Emerg. Microbes Infect..

[18] Ramilo O (2007). Gene expression patterns in blood leukocytes discriminate patients with acute infections. Blood.

[19] Lee YS (2005). Molecular signature of clinical severity in recovering patients with severe acute respiratory syndrome coronavirus (SARS-CoV). BMC Genomics.

[20] Subramanian A (2005). Gene set enrichment analysis: a knowledge-based approach for interpreting genome-wide expression profiles. Proc. Natl Acad. Sci. USA.

[21] Pugin J (1991). Diagnosis of ventilator-associated pneumonia by bacteriologic analysis of bronchoscopic and nonbronchoscopic “blind” bronchoalveolar lavage fluid. Am. Rev. Respir. Dis..

[22] Hoshida Y (2010). Nearest template prediction: a single-sample-based flexible class prediction with confidence assessment. PLoS ONE.

[23] Rutkowska-Zapala M (2015). Human monocyte subsets exhibit divergent angiotensin I-converting activity. Clin. Exp. Immunol..

[24] Nikitina, E., Larionova, I., Choinzonov, E. & Kzhyshkowska, J. Monocytes and macrophages as viral targets and reservoirs. *Int. J. Mol. Sci.* **19**, 10.3390/ijms19092821 (2018). 10.3390/ijms19092821 PMC6163364 30231586

[25] Giamarellos-Bourboulis EJ (2020). Complex immune dysregulation in COVID-19 patients with severe respiratory failure. Cell Host Microbe.

[26] Ottestad, W., Seim, M. & Mæhlen, J. O. COVID-19 with silent hypoxemia. *Tidsskr Nor Laegeforen* **140**, 10.4045/tidsskr.20.0299 (2020). 10.4045/tidsskr.20.0299 32378842

[27] Poissy J (2020). Pulmonary embolism in COVID-19 patients: awareness of an increased prevalence. Circulation.

[28] Jassal B (2020). The reactome pathway knowledgebase. Nucleic Acids Res..

[29] Esmon CT, Xu J, Lupu F (2011). Innate immunity and coagulation. J. Thromb. Haemost..

[30] Mathian, A. & Amoura, Z. Response to: ‘Are patients with systemic lupus erythematosus at increased risk for COVID-19?’ by Favalli et al. *Ann. Rheum. Dis.*, 10.1136/annrheumdis-2020-217859 (2020). 10.1136/annrheumdis-2020-217859 32451345

[31] Mathian, A. et al. Clinical course of coronavirus disease 2019 (COVID-19) in a series of 17 patients with systemic lupus erythematosus under long-term treatment with hydroxychloroquine. *Ann. Rheum. Dis*., 10.1136/annrheumdis-2020-217566 (2020). 10.1136/annrheumdis-2020-217566 32332072

[32] Kell AM, Gale M (2015). RIG-I in RNA virus recognition. Virology.

[33] Lorizate M, Krausslich HG (2011). Role of lipids in virus replication. Cold Spring Harb. Perspect. Biol..

[34] Rooney MS, Shukla SA, Wu CJ, Getz G, Hacohen N (2015). Molecular and genetic properties of tumors associated with local immune cytolytic activity. Cell.

[35] Grifoni A (2020). Targets of T cell responses to SARS-CoV-2 coronavirus in humans with COVID-19 disease and unexposed individuals. Cell.

[36] Wu C, Jin X, Tsueng G, Afrasiabi C, Su AI (2016). BioGPS: building your own mash-up of gene annotations and expression profiles. Nucleic Acids Res..

[37] Ledford H (2020). How does COVID-19 kill? Uncertainty is hampering doctors’ ability to choose treatments. Nature.

[38] Gandhi M, Yokoe DS, Havlir DV (2020). Asymptomatic transmission, the Achilles’ heel of current strategies to control Covid-19. N. Engl. J. Med.

[39] Liu Y (2020). Neutrophil-to-lymphocyte ratio as an independent risk factor for mortality in hospitalized patients with COVID-19. J. Infect..

[40] Wang T (2020). Attention should be paid to venous thromboembolism prophylaxis in the management of COVID-19. Lancet Haematol..

[41] Jose, R. J. & Manuel, A. COVID-19 cytokine storm: the interplay between inflammation and coagulation. *Lancet Respir. Med.*, 10.1016/S2213-2600(20)30216-2 (2020). 10.1016/S2213-2600(20)30216-2 PMC7185942 32353251

[42] Tay MZ, Poh CM, Renia L, MacAry PA, Ng LFP (2020). The trinity of COVID-19: immunity, inflammation and intervention. Nat. Rev. Immunol..

[43] Garcia-Beltran WF (2016). Open conformers of HLA-F are high-affinity ligands of the activating NK-cell receptor KIR3DS1. Nat. Immunol..

[44] Zhang, D. et al. COVID-19 infection induces readily detectable morphological and inflammation-related phenotypic changes in peripheral blood monocytes. *J. Leukoc. Biol*. (2020). 10.1002/JLB.4HI0720-470R PMC7675546 33040384

[45] Zhou, Y. et al. Pathogenic T-cells and inflammatory monocytes incite inflammatory stormsin severe COVID-19 patients. *Natl Sci. Rev*. **7**, 998–1002 (2020). 10.1093/nsr/nwaa041 PMC7108005 34676125

[46] Cao X (2020). COVID-19: immunopathology and its implications for therapy. Nat. Rev. Immunol..

[47] Parker D (2018). A live vaccine to Staphylococcus aureus infection. Virulence.

[48] Bachanova V (2020). Chimeric antigen receptor T cell therapy during the COVID-19 pandemic. Biol. Blood Marrow Transpl..

[49] Gautier L, Cope L, Bolstad BM, Irizarry R (2004). A. affy-analysis of Affymetrix GeneChip data at the probe level. Bioinformatics.

[50] Davis S, Meltzer PS (2007). GEOquery: a bridge between the Gene Expression Omnibus (GEO) and BioConductor. Bioinformatics.

[51] Tusher VG, Tibshirani R, Chu G (2001). Significance analysis of microarrays applied to the ionizing radiation response. Proc. Natl Acad. Sci. USA.

[52] Schwender, H. *siggenes: Multiple Testing using SAM and Efron’s Empirical Bayes Approaches*. R package version 1.62.0 (R Foundation for Statistical Computing, 2020).

[53] Collisson EA (2011). Subtypes of pancreatic ductal adenocarcinoma and their differing responses to therapy. Nat. Med..

[54] Sadanandam A (2013). A colorectal cancer classification system that associates cellular phenotype and responses to therapy. Nat. Med..

[55] Sadanandam A (2015). A cross-species analysis in pancreatic neuroendocrine tumors reveals molecular subtypes with distinctive clinical, metastatic, developmental, and metabolic characteristics. Cancer Discov..

[56] Reich M (2006). GenePattern 2.0. Nat. Genet.

[57] Federico A, Monti S (2020). hypeR: an R package for geneset enrichment workflows. Bioinformatics.

[58] Yu G, Wang LG, Yan GR, He QY (2015). DOSE: an R/Bioconductor package for disease ontology semantic and enrichment analysis. Bioinformatics.

[59] Chen EY (2013). Enrichr: interactive and collaborative HTML5 gene list enrichment analysis tool. BMC Bioinformatics.

[60] Kanehisa M, Furumichi M, Tanabe M, Sato Y, Morishima K (2017). KEGG: new perspectives on genomes, pathways, diseases and drugs. Nucleic Acids Res..

[61] Binder JX (2014). COMPARTMENTS: unification and visualization of protein subcellular localization evidence. Database (Oxf.).

[62] Pinero J (2017). DisGeNET: a comprehensive platform integrating information on human disease-associated genes and variants. Nucleic Acids Res..

