# Supplementary material for: A blood transcriptome-based analysis of disease progression, immune regulation, and symptoms in coronavirus-infected patients
Source: Cell Death Discov. 2020 Dec 8;6:141. doi: 10.1038/s41420-020-00376-x (PMC7721861; doi:10.1038/s41420-020-00376-x)
Supplement: Supplementary file 1 — Supplementary Information [file 41420_2020_376_MOESM1_ESM.docx]

**A blood transcriptome-based analysis of disease progression, immune regulation** **and symptoms in coronavirus-infected patients**

**Supplementary Information**

**Supplementary Figure Legends**

**Supplementary Figure 1. SAM analysis output for CoV gene signature identification.** A) Delta vs. FDR plot. B) Delta vs. Significant genes. C) SAM plot showing significant differentially expressed genes.

**Supplementary Figure 2. PBMC show association with bacterial and other viral infections.** CoV-Up-gene scores (A) and CoV-Down-gene scores (B) and their association with patients affected by different bacterial and viral infections and healthy individuals. Transcriptome data for 144 samples for this analysis was from Ramilo *et al^22^*. Kruskal-Wallis statistical with nominal p < 0.0001.

**Supplementary Figure 3. Enrichment of PBMC from other diseases.** A) Enrichment analysis of CoV-Up-gene signatures using MSigDB’s C7 immune gensets.

**Supplementary Table Legends**

**Supplementary Table 1. A.** SAM analysis and differential gene expression in PBMC between SARS patients and healthy individuals. **B.** Differential expression of genes in PBMC between SARS patients and healthy individuals from SAM analysis. **C.** ssGSEA analysis of PBMC from COVID-19 patients and healthy individuals. **D.** ssGSEA analysis of PBMC from SARS (acute vs. recovering) and other infection patients and healthy individuals. **E.** ssGSEA analysis of PBMC from bacterial and viral infections and healthy individuals. **F.** NTP analysis of acute-like vs. recovering-like SARS patients and healthy volunteers.

**Supplementary Table 2. A.** Hypergeometric enrichment analysis using MSigDB's hallmarks database and hypeR package. **B.** Hypergeometric enrichment analysis using REACTOME database and hypeR package. **C.** Hypergeometric enrichment analysis using KEGG database and hypeR package. **D.** Hypergeometric enrichment analysis using subcellular localization COMPARTMENTS database and hypeR package. **E.** ssGSEA analysis of Rooney et al. immune genes on SARS patient samples. **F.** ssGSEA analysis of Rooney et al. immune genes on COVID-19 patient samples. **G.** Hypergeometric enrichment analysis using MSigDB's C7 database and hypeR package. **H.** Hypergeometric enrichment analysis using BioGPS UP database and hypeR package. **I.** Hypergeometric enrichment analysis using BioGPS Down database and hypeR package. **J.** Hypergeometric enrichment analysis using DGN database and DOSE package.
