# Supplementary figures and images for: A blood transcriptome-based analysis of disease progression, immune regulation, and symptoms in coronavirus-infected patients
Source: Cell Death Discov. 2020 Dec 8;6:141. doi: 10.1038/s41420-020-00376-x (PMC7721861; doi:10.1038/s41420-020-00376-x)

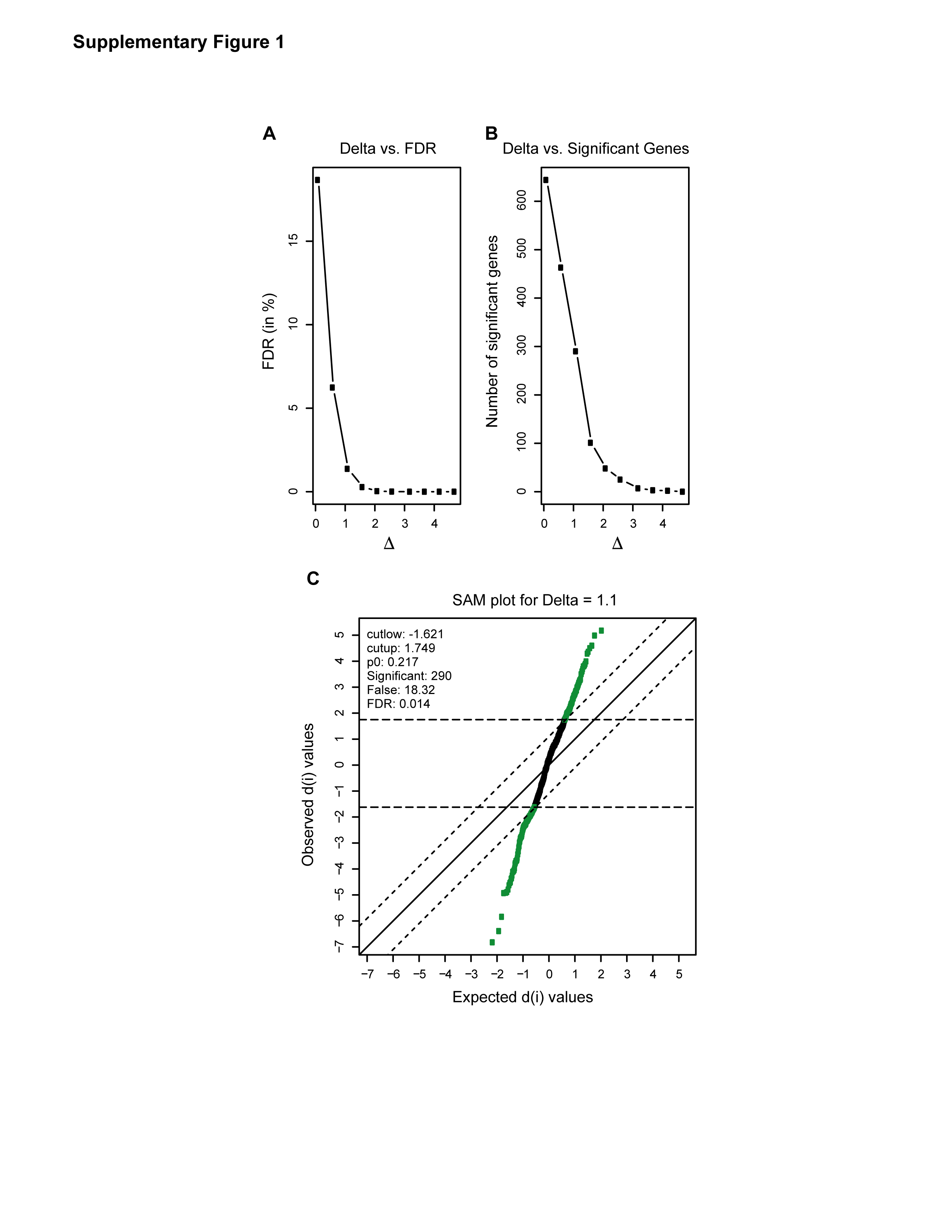

Supplement: Supplementary file 2 — Supplementary Figure 1 [file 41420_2020_376_MOESM2_ESM.tif]

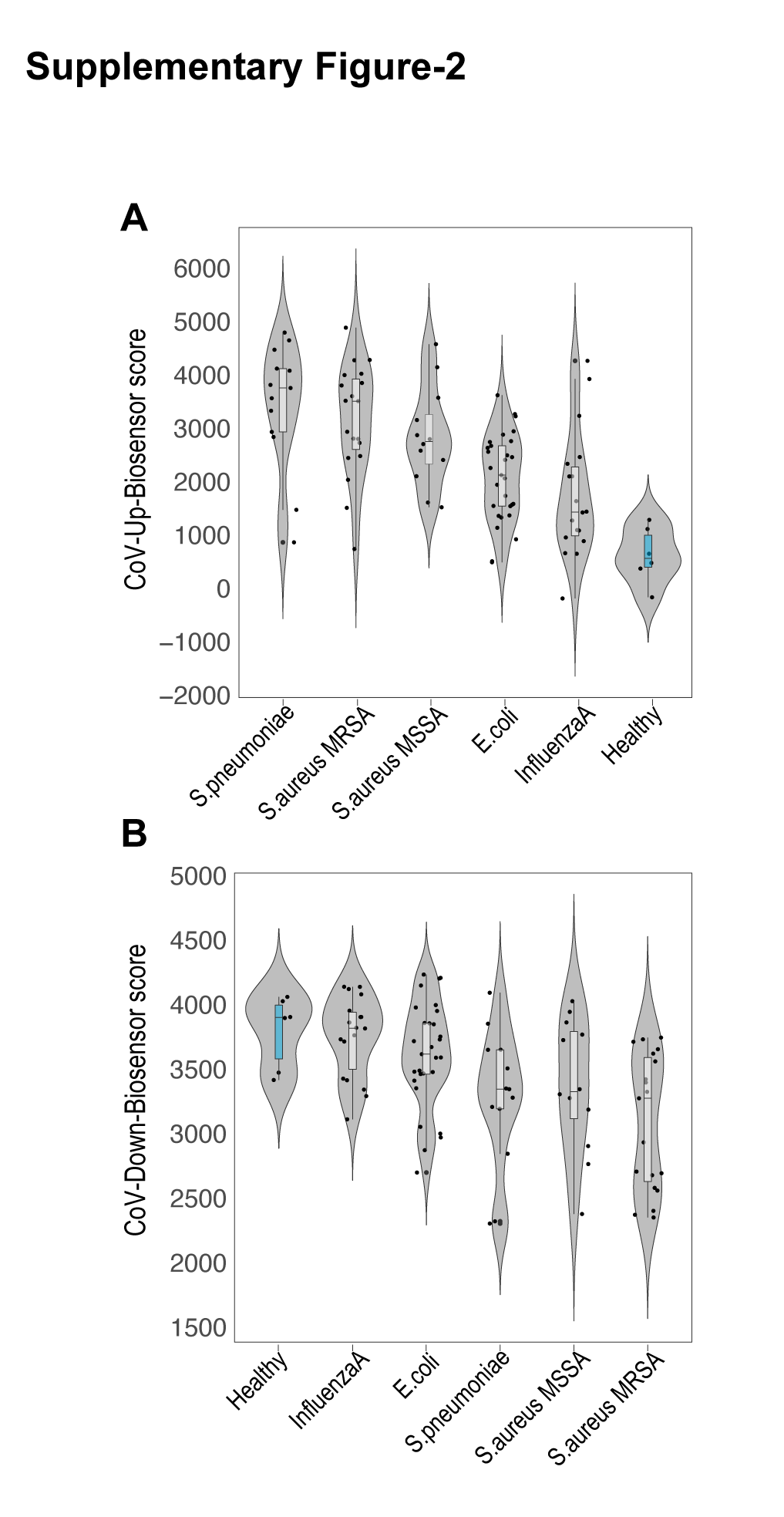

Supplement: Supplementary file 3 — Supplementary Figure 2 [file 41420_2020_376_MOESM3_ESM.tif]

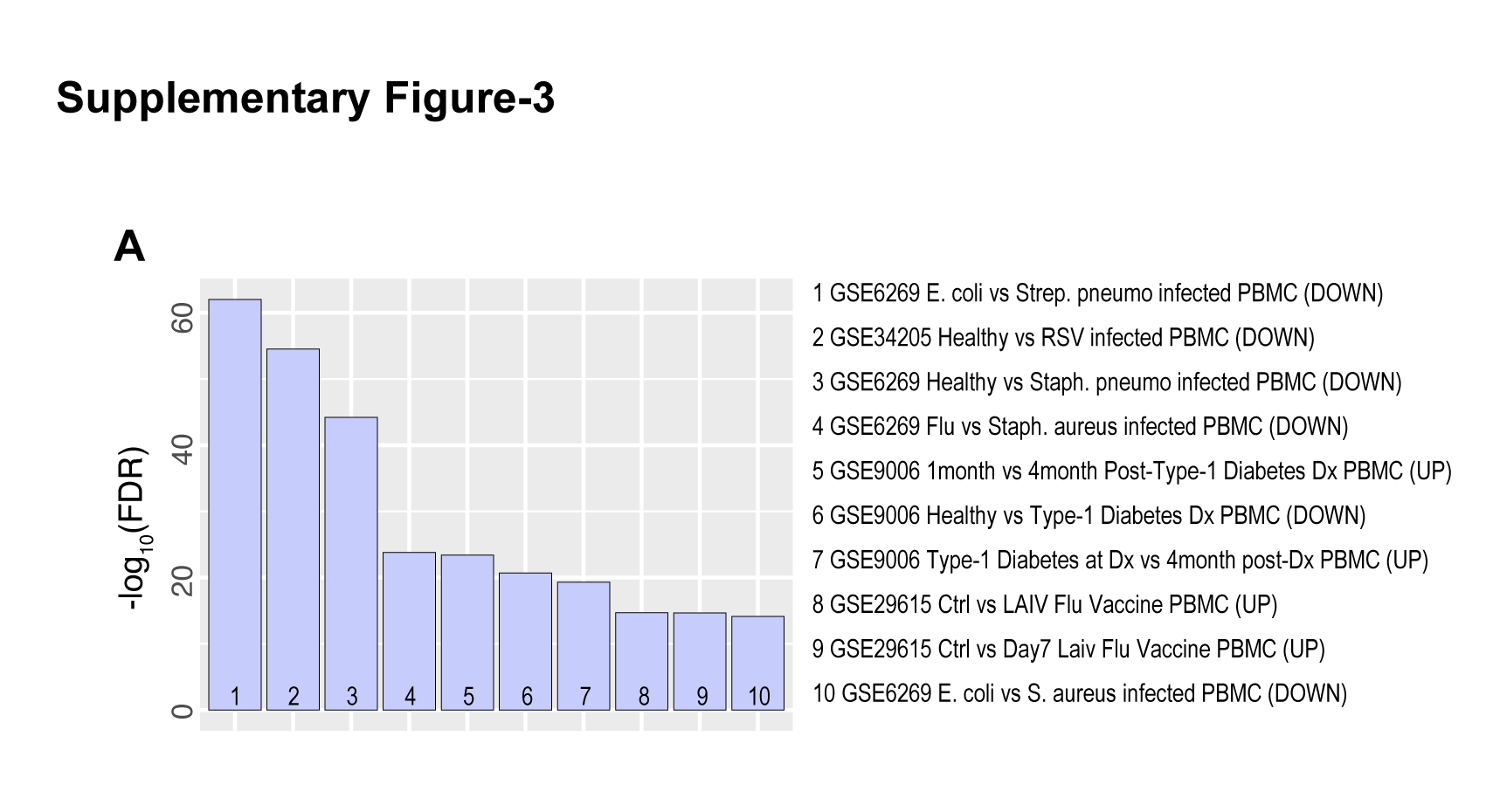

Supplement: Supplementary file 4 — Supplementary Figure 3 [file 41420_2020_376_MOESM4_ESM.tif]
